# Morphogens in the evolution of size, shape and patterning

**DOI:** 10.1242/dev.202412

**Published:** 2024-09-18

**Authors:** L.S. Mosby, A.E. Bowen, Z. Hadjivasiliou

**Affiliations:** 1 The Francis Crick Institute: Mathematical and Physical Biology Laboratory, 1 Midland Road, London, NW1 1AT, UK; 2 University College London: Department of Physics and Astronomy, Gower Street, London, WC1E 6BT, UK; 3 London Centre for Nanotechnology, 19 Gordon Street, London, WC1H 0AH, UK

## Abstract

Much of the striking diversity of life on Earth has arisen from variations in the way that the same molecules and networks operate during development to shape and pattern tissues and organs into different morphologies. However, we still understand very little about the potential for diversification exhibited by different, highly conserved mechanisms during evolution, or, conversely, the constraints that they place on evolution. With the aim of steering the field in new directions, we focus on morphogen-mediated patterning and growth as a case study to demonstrate how conserved developmental mechanisms can adapt during evolution to drive morphological diversification and optimise functionality, and to illustrate how evolution algorithms and computational tools can be used alongside experiments to provide insights into how these conserved mechanisms can evolve. We first introduce the conserved properties of morphogen-driven patterning mechanisms, before summarising comparative studies that exemplify how changes in the spatiotemporal expression and signalling levels of morphogens impact the diversification of organ size, shape and patterning in nature. Finally, we detail how theoretical frameworks can be used in conjunction with experiments to probe the role of morphogen-driven patterning mechanisms in evolution. We conclude that morphogen-mediated patterning is an excellent model system and offers a generally applicable framework for the investigation of the evolution of developmental mechanisms.

## Introduction

Morphogens regulate patterning and growth throughout development and evolution, and the same morphogen families are used in varying developmental contexts, at different orders of magnitude, and across species (Madamanchi et al., 2021). Extensive study over the past decades has led to a solid understanding of the versatile mechanisms through which morphogens regulate cell response and tissue-level patterning, and has generated quantitative theoretical frameworks for morphogen gradient formation, patterning and growth that recapitulate experimental observations (for recent reviews see (Kicheva & Briscoe, 2023; Stapornwongkul & Vincent, 2021)). Despite these advances, we have a much more fragmentary picture of the role morphogens play in evolution. In this Review, we discuss conserved properties of morphogen-driven patterning and the evolutionary trade-offs they entail, before summarising comparative studies that illustrate how spatial and temporal properties of morphogen signalling can be modified to affect the evolution of patterning and form across animal species. We do not discuss morphogen-mediated patterning in plants here, but we refer readers to relevant reviews on this topic (Klesen et al., 2020). Finally, we discuss how theoretical approaches can be combined with experiments to achieve a deeper, more mechanistic understanding of the evolution of morphogen-driven mechanisms and development more broadly.

## Conserved properties of morphogen-driven patterning

**Figure 1:**
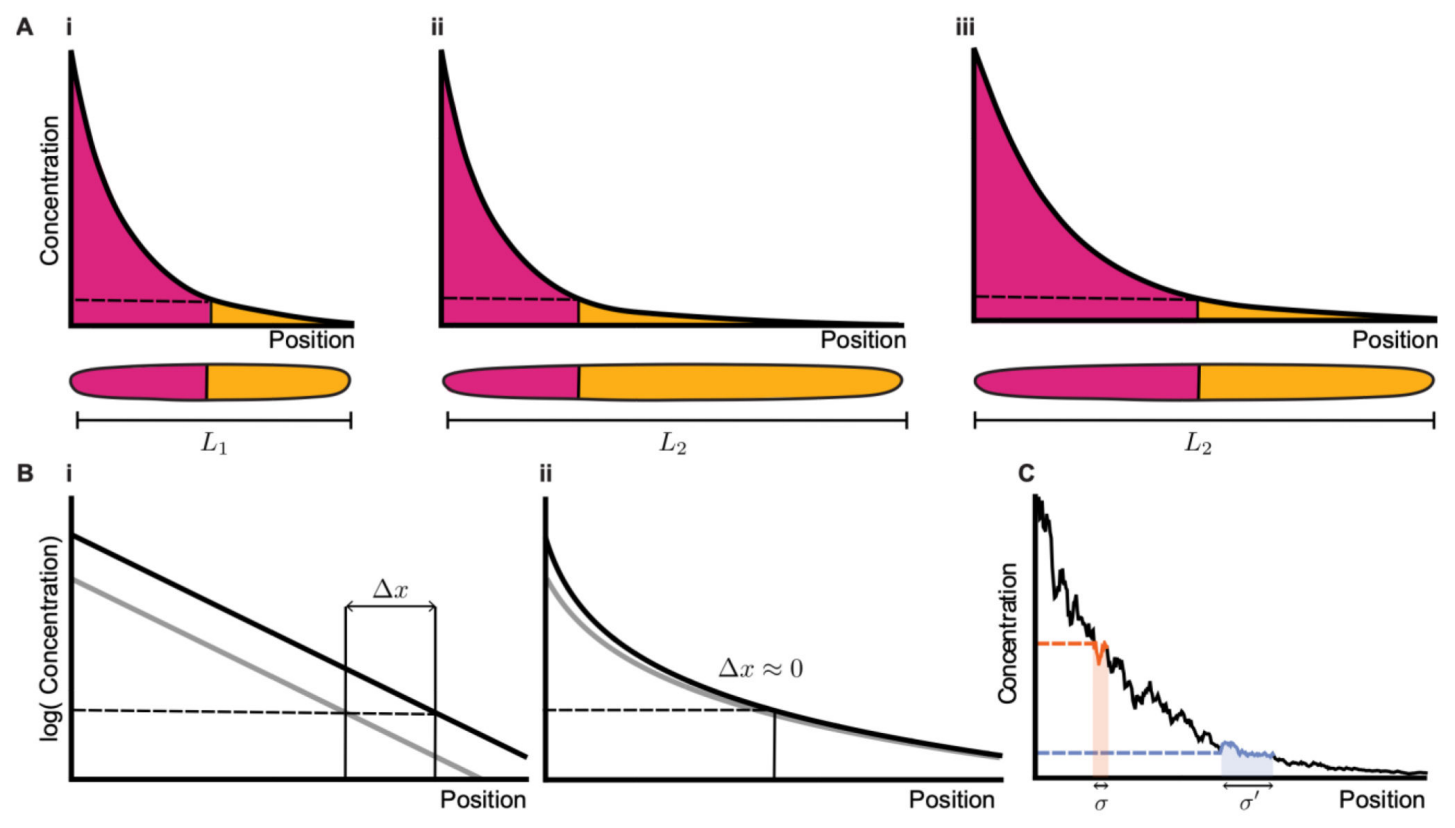
Conserved properties of morphogen-mediated patterning A: Morphogens can pattern tissues in a concentration-dependent manner. (i) In the example shown, a local concentration above or below a threshold (dashed line) results in differentiation into different cell types (pink, yellow). This results in tissue patterning (below). (ii) When tissue size changes (from L_1_ in i to L_2_ in ii) but morphogen gradients do not scale, the proportions of the tissue pattern are distorted. In this example, the morphogen gradient remains completely unchanged following an increase in tissue length, reflected in an increase in the absolute size of only one cell type (yellow) and distortion of the pattern (relative size of pink or yellow regions). (iii) When the morphogen gradient scales, the boundaries that define cell types move proportionally to the tissue size, and pattern proportions are maintained when size changes. B: (i) The morphogen gradient in log scale for baseline (grey) and increased (black) production rates. A non-robust morphogen gradient exhibits a large shift Δx in the position of the cell type boundary it defines at a given concentration threshold (dashed line) following a shift in system parameters such as morphogen production. This is indicated by the same threshold in concentration (dashed line) reaching significantly different positions for the morphogen gradients shown in grey and black. (ii) Robust morphogen gradients can buffer changes in morphogen production so that cell type boundaries do not change in a target region far enough from the morphogen source. This corresponds to a value of Δx close to zero. C: A noisy morphogen gradient (black) and the positional errors corresponding to two specific concentration thresholds close to the morphogen source where the gradient is steep (positional error *σ*; orange), and further away where the gradient is shallow (positional error *σ*′; blue). The steeper the morphogen profile, the smaller the positional error and the more precise the morphogen readout.

Morphogen gradients adapt to variation in size, termed scaling, and to genetic and environmental perturbations, often referred to as robustness (Fig. 1A, B). In addition, cell fate boundaries specified downstream of morphogens are extremely precise despite high levels of noise (Fig. 1C). Scaling, robustness and precision are important in several developmental contexts because they buffer intrinsic and extrinsic noise and perturbations, resulting in remarkably reproducible developmental patterning and size which is key for embryo viability and adult fitness. In this section, we discuss morphogen scaling, robustness and precision with a focus on evolution. We provide a comparative perspective on mechanisms that drive these properties, consider ways in which they may constrain or facilitate morphological diversification, and discuss evolutionary trade-offs between them.

### Scaling

Morphogen scaling during development is important for maintaining proportionate patterning in the face of natural variation in size between individuals of the same species (Fig. 1A). Morphogen scaling can occur during developmental growth, with examples including the dynamic scaling of the Dpp morphogen gradient during *Drosophila* eye and wing development (Hamaratoglu et al., 2011; Wartlick et al., 2011, 2014) and the scaling of Bone Morphogenetic Protein (Bmp) signalling with pectoral fin size in the developing zebrafish (Mateus et al., 2020). The expression domains and levels of Wnts, Bmps and their respective repressors scale with the size of *Xenopus* embryos (Leibovich et al., 2020). Experimentally size-reduced zebrafish embryos show that somite size scaling can be explained by the scaling of the associated Fgf and Wnt gradients (Ishimatsu et al., 2018). Similarly, the gradients of Nodal and its repressor Lefty, which pattern the germ layers, and the Bmp gradient, which patterns the dorso-ventral (DV) axis, also adjust to zebrafish embryo size (Almuedo-Castillo et al., 2018; Huang & Umulis, 2019). In fact, zebrafish embryos that were reduced in size by up to 30% before gastrulation regain correct proportions and morphogen scaling within two hours, suggesting that the feedback regulating morphogen scaling is fast (Almuedo-Castillo et al., 2018; Huang & Umulis, 2019). These data highlight the prevalence of morphogen scaling during developmental patterning. However, more information is needed as to how this scaling is achieved, how conserved such scaling mechanisms might be between species, and what role morphogen scaling plays in the evolution of patterning. Proposed mechanisms of morphogen scaling range from scaling of the morphogen gradient amplitude or source size (Ho et al., 2024; Umulis & Othmer, 2012) and dilution-dependent mechanisms (Aguilar-Hidalgo et al., 2018; Averbukh et al., 2014; Fried & Iber, 2014) to scaling mediated by feedback between morphogens and other molecules (Ben-Zvi & Barkai, 2010; Ben-Zvi et al., 2008). Experimental evidence primarily exists for the latter, so below we highlight feedback-based mechanisms that are present across evolution.

The expansion-repression model proposes that morphogen scaling is achieved through interactions between morphogen and diffusible ‘expander’ molecules (Ben-Zvi & Barkai, 2010). Here, the expander can either inhibit the degradation or enhance the diffusion of the morphogen to increase its range, while morphogen signalling represses expander production. A few systems resemble the dynamics predicted by the expansion-repression model: for example, the Dpp morphogen gradients in the *D. melanogaster* wing and eye imaginal discs scale through interactions with the diffusible molecule Pentagone (Pent) (Romanova-Michaelides et al., 2022; Vuilleumier et al., 2010; Wartlick et al., 2014). In the absence of Pent, Dpp scaling fails, leading to patterning and growth defects, whereas Pent over-expression causes the morphogen gradient to over-expand (Ben-Zvi et al., 2011). Similar properties have been demonstrated for Smoc proteins that interact with Bmp in *Xenopus* embryos (Thomas et al., 2017) and in the zebrafish pectoral fin (Mateus et al., 2020). A similar mechanism might also explain the scaling behaviour of Nodal and Lefty in zebrafish embryos (Almuedo-Castillo et al., 2018), and how Sonic Hedgehog (Shh) scales in the zebrafish neural tube via interactions with Scube2 (Collins et al., 2023). Together, these results indicate that expander molecules and similar feedback mechanisms represent a conserved apparatus for morphogen scaling across species.

Alternatively, scaling can be achieved using a shuttling mechanism (Ben-Zvi et al., 2008). Morphogen shuttling occurs during DV patterning in *Drosophila* and *Xenopus* embryos through interactions between Bmp morphogens and Bmp binding proteins/inhibitors that prevent Bmp signalling (such as Chordin and Sog) (Ben-Zvi et al., 2008; Eldar et al., 2002). These interactions generate complexes that exhibit enhanced diffusion and degradation compared to the dynamics of the free ligands. The inhibitors also act as chaperones, generating a flux of morphogens towards domains where inhibitors are absent, namely the morphogen source region. Scaling is achieved in *Xenopus* embryos when the different Bmp morphogens that are expressed dorsally and ventrally exhibit different binding affinities for the Bmp inhibitors, and when the dorsally-expressed Admp is repressed by Bmp Signalling (Ben-Zvi et al., 2008).

In the context of morphological diversification, a question that emerges is how the presence of scaling mechanisms affects the evolution of novel size-pattern proportions. In principle, feedback-mediated scaling implies that changes in organ size will always be accompanied by proportional adaptation in patterning, as observed for example in the case of Bcd scaling across fly species (Gregor et al., 2005). This would be expected to constrain the range of potential phenotypes and inhibit diversification. Nonetheless, modulation in the parameters that regulate feedback between size and pattern may lead to new types of pattern whilst maintaining scaling properties between individuals of the same species. Going forward, this can be rigorously explored using theoretical tools that probe the phase-space of scaling patterns and size-shape covariance for systems exhibiting morphogen-mediated patterning and growth. Experimentally, quantification of the standing variation in size and pattern, and their covariance in the presence and absence of morphogen scaling within and across species, can help to uncover constraints that scaling mechanisms place on size and pattern evolution.

### Robustness

Heterozygous *Drosophila* embryos that produce half of the Bmp homolog Screw, the Bmp inhibitor Sog, or the Sog protease Tld, generate the same dorsal patterning phenotype as wild-type embryos (Eldar et al., 2002), and mostly wild-type patterning is achieved during the development of the *Drosophila* wing in heterozygous Hedgehog (Hh) mutants (Irons et al., 2010). Similar experiments in vertebrates have shown that zebrafish embryos that are heterozygous for *lft1* and *lft2*, or are *lft1* knock-outs, exhibit nearly wild-type Nodal levels and germ layer specification (Almuedo-Castillo et al., 2018). Together, these data demonstrate that morphogen patterning is robust to changes in the copy number of genes encoding for the production of morphogens or their inhibitors.

Robustness to perturbations in morphogen production relies heavily on the decay of the morphogen gradient close to the source region; if changes in production are buffered close to the source, then gene expression boundaries specified in a target region far from the source will remain invariant to these perturbations (Eldar et al., 2003; Irons et al., 2010; Fig. 1B). This can be achieved through self-enhanced morphogen degradation that selectively increases degradation local to the morphogen source (Eldar et al., 2003). Known examples of self-enhanced morphogen degradation include Wingless (Wg) and Hh in the *D. melanogaster* wing disc (Chen & Struhl, 1996; Eldar et al., 2003; Irons et al., 2010; Reyes et al., 2022), retinoic acid during development of the zebrafish nervous system (White et al., 2007) and Shh in the vertebrate neural tube (Balaskas et al., 2012; Dessaud et al., 2007). Self-enhanced degradation is typically mediated through feedback that increases receptor production as a response to increased morphogen levels (Chen & Struhl, 1996; Dessaud et al, 2007; Reyes et al., 2022; White et al., 2007).

Robustness can also be achieved through feedback that directly regulates ligand transport as a function of signalling levels. An example is the induction-contraction mechanism, present during DV axis patterning in the *Drosophila* embryo (Rahimi et al., 2016). Here, Toll receptor activation generates a gradient of the transcription factor Dl that ultimately patterns the embryo DV axis. Intermediate levels of Toll signalling induce the expression of WntD, which inhibits Toll receptor activation and reduces the decay length of the Dl gradient. This feedback can reverse fluctuations in the Dl gradient decay length by effectively ‘pinning’ the Dl concentration at a specified position. A similar feedback loop operates between Nodal and Lefty in zebrafish, where Nodal signalling induces the production of Lefty molecules that then inhibit Nodal-receptor interactions (Rogers et al., 2017). In this case, the feedback mechanism is not necessary for normal development, but mitigates the effects of perturbations in Nodal signalling levels. Shuttling-based feedback is another mechanism that conveys robustness to perturbations in morphogen production in *Drosophila* (Eldar et al., 2002; Haskel-Ittah et al., 2012), *Xenopus* (Ben-Zvi et al., 2008) and *Tribolium* (Zee et al., 2006) embryos, primarily by ‘storing’ morphogen molecules near their source where shuttling molecules are absent (for a review see (Shilo & Barkai, 2017)). This does not influence cell fate decisions for regions with high morphogen concentration, but it buffers fluctuations in morphogen concentration further away from the morphogen source as long as the morphogen decays sufficiently fast. The induction-contraction and shuttling mechanisms were proposed to work collaboratively to increase the robustness of the Toll pathway during DV patterning in the *Drosophila* embryo (Haskel-Ittah et al., 2012; Rahimi et al., 2016). This is an example of functional redundancy, where several mechanisms that mediate robustness are at play simultaneously.

Robustness may also be conferred by redundancies in the Gene Regulatory Networks (GRNs) that interpret the morphogen gradients. As well as primary enhancer regions that are usually ‘switched on’ by morphogens, gene expression can also be regulated by secondary ‘shadow’ enhancer regions that interact with alternative/downstream signalling molecules (Frankel et al., 2010; Perry et al., 2010). The overlapping expression patterns generated by primary and shadow enhancers are important for ensuring that transcription levels are maintained in heterozygous mutants for *dl* during DV axis patterning (Perry et al., 2010), and for *wg* during quaternary trichome development (Frankel et al., 2010) in *Drosophila*. In contrast, species such as the insect *Tribolium castaneum* that do not possess redundant Bmp inhibitors lose their entire central nervous system following the loss of *Tc-sog* which encodes for the only morphogen inhibitor (Zee et al., 2006).

### Precision

Morphogen-mediated patterning leads to sharp gene expression boundaries (Fig. 1C); the boundaries that separate cell types have a very small associated width, sometimes as small as the width of a single cell (Bollenbach et al., 2008; Gregor et al., 2007; Zagorski et al., 2017). Temporal or spatial averaging improves precision (Gregor et al., 2007), and spatial smoothing through diffusion suppresses noise in the morphogen gradient (Bollenbach et al., 2008).

Most mechanisms found to increase precision implicate the GRNs downstream of morphogens (recently reviewed in (Kicheva & Briscoe, 2023)). A classic example is the case of Hunchback (Hb), which rapidly forms a sharp boundary in response to Bcd signalling with a precision in the boundary position estimated at □ 4% of the embryo length (Morton de Lachapelle & Bergmann, 2010; Nikolić et al., 2023; Perry et al., 2012). This precision may be achieved via co-operative binding of Bcd to target DNA sequences such as *hb* promoters and enhancers (Gregor et al., 2007; Lopes et al., 2012), or through Hb self-activation (Lopes et al., 2008, 2012), both of which can increase the steepness of the Hb profile. The GRN downstream of Shh in the mouse ventral neural tube also impacts precision: modifying the regulatory dynamics of the GRN alters the sensitivity of the stochastic switching rates between cell fates to changes in Shh concentration (Exelby et al., 2021). Similarly, retinoic acid sets up the initially noisy gene expression of h*oxb1a* and *krox20* in the zebrafish hindbrain, which then sharpen their own expression boundaries through mutual self-activation and cross-inhibition (L. Zhang et al., 2012).

The generation of sharp and precise boundaries from a gradually decaying morphogen gradient is a key feature of the mutual activation and inhibition typically encoded in GRNs. This means that GRNs downstream of morphogens hold a central role in pattern determination, and so are obvious candidates for impacting pattern evolution. Indeed, pivotal events in evolution like the fin-to-limb transition have been ascribed, at least in part, to changes in the strength of interactions in conserved GRNs downstream of morphogens (Onimaru et al., 2016). In addition, theoretical studies and recent work using synthetic GRNs to explore pattern evolution downstream of morphogens are shedding light on the role of GRNs in pattern diversification and evolvability. For example, properties like modularity in network design were shown to aid pattern evolvability (Verd et al., 2019), and the non-linearity emerging from feedback between transcription factors in GRNs leads to epistatic effects that could influence the genotype-to-phenotype map and evolutionary paths (Baier et al., 2023; Santos-Moreno et al., 2023).

### Design principles and trade-offs

Two questions that follow from this discussion are: how the feedback and network topologies that drive morphogen scaling, robustness and precision have evolved; and whether trade-offs between them exist. For example, robustness generally requires fast morphogen decay and steep morphogen gradients near the morphogen source, but this results in a gradient that becomes flat over relatively short length scales, impairing precision (Adelmann et al., 2023). The trade-off between robustness and precision has been quantified through the ‘useful patterning region’ of a morphogen gradient, in which a morphogen gradient can simultaneously generate robust and precise gene expression boundaries (Lander et al., 2009; Lo et al., 2015). Patterning far from the source region requires sacrificing the width of the useful patterning region, which offers one explanation for why anti-parallel morphogen gradients are often necessary to generate precise and robust patterns across the entire length of a tissue (Zagorski et al., 2017).

An example of the trade-off between robustness and precision is evident during anterior-posterior (AP) axis formation in the *Drosophila* wing imaginal disc, when expression domains of the transcription factor Col and the morphogen Dpp are established at different times during the formation of the Hh gradient with the aim of increasing robustness or precision respectively (Reyes et al., 2022). In this system, the steady-state Hh gradient is robust to changes in morphogen production as a result of Hh-dependent up-regulation of the Hh receptor Ptch, resulting in self-enhanced degradation (Chen & Struhl, 1996; Reyes et al., 2022). Since the boundary of Col expression near the Hh source is established after the Hh gradient reaches steady state, it exhibits enhanced robustness (Reyes et al., 2022). In contrast, the Dpp source is established during a transient overshoot of the Hh gradient above its steady-state and before Hh receptor up-regulation commences, when the Hh gradient is steeper, improving precision instead (Reyes et al., 2022).

In theory, increasing the amplitude of the morphogen gradient improves read-out precision (Gregor et al., 2007; Song & Hyeon, 2021). This has also been linked to improved robustness in theoretical models that include the saturation of non-signalling receptors, potentially bypassing trade-offs between them (Irons et al., 2010). However, the production of more molecules increases the associated metabolic costs of transcription, translation and degradation (Song & Hyeon, 2021; Szekely et al., 2013). This putative cost-precision trade-off instructs that for a given gene expression boundary position there exists an optimal morphogen gradient decay length that jointly minimises cost and maximises precision (Song & Hyeon, 2021). According to this model, the gradients of Bcd, Wg, Hh and Dpp in the *Drosophila* embryo and imaginal discs appear to operate at decay lengths that result in a near-optimal trade-off (Song & Hyeon, 2021), suggesting that metabolic costs may counterbalance the need for extremely precise gene expression boundaries. Note, however, that living systems operate far from equilibrium, and the extent to which the evolution of multicellular animals is limited by metabolic costs at the cellular level is not clear.

In some instances, the feedback mechanisms that mediate scaling and robustness appear to function antagonistically. For example, self-repressed degradation facilitates scaling, whereas self-enhanced degradation promotes robustness (Eldar et al., 2003; Lander et al., 2009; Y. Zhu et al., 2020). Although shuttling mechanisms have been implicated in facilitating both scaling and robustness (Ben-Zvi et al., 2008; Eldar et al., 2002), a comprehensive picture of the conditions that facilitate both scaling and robustness, their trade-offs, or the possible evolutionary paths that optimise both properties are lacking.

A common feature in many morphogen-driven systems is the presence of both signalling and non-signalling receptors (X. Lin, 2004; Stapornwongkul et al., 2020). In principle, this decouples signalling sensitivity from ligand transport and turnover, and could explain the presence of promiscuous, non-signalling receptors such as heparan sulphate proteoglycans (HSPGs) in the Bmp, Wnt, Hh, and Fgf pathways (X. Lin, 2004; Stapornwongkul & Vincent, 2021). This ‘design’ that combines signalling and non-signalling receptors can also promote precise and robust morphogen read-out (Iyer et al., 2023; Lo et al., 2015). In addition, many of the mechanisms that mediate scaling and robustness act primarily to regulate the dispersal or degradation rate of morphogen ligands by adjusting interactions with non-signalling receptors (Iyer et al., 2023; Romanova-Michaelides et al., 2022). The ways in which signalling and non-signalling receptors impact the evolvability of morphogen-driven patterning or the route through which they evolved are not well understood, but would be interesting to explore in theoretical studies and comparative analyses.

A final trade-off to consider is between the properties of morphogen signalling and pattern evolvability. For example, robustness is often associated with reduced evolvability because it implies that the phenotype is invariant to genetic perturbations. In the context of GRNs, properties that facilitate this robustness, such as network redundancy and modularity (Frankel et al., 2010; Perry et al., 2010; Hernández et al., 2022), allow neutral mutations to accumulate that do not affect fitness but which can then be suddenly released akin to a ‘tipping point’ (Hallgrímsson et al., 2023; Levy & Siegal, 2008). More broadly, theoretical work indicates that certain physical constraints can result in the emergence of distinct ‘slow modes’ along which a high-dimensional system can exhibit phenotypic plasticity, while remaining robust to other changes (Husain & Murugan, 2020; Kaneko, 2024). This effective dimensional reduction can accelerate evolution by inducing correlated mutational changes in multiple parameters. A similar slow mode is present in scaling systems, such that perturbations in tissue length only induce changes to system parameters along this mode (Nikolić et al., 2023), which indicates that scaling mechanisms maintain robustness to changes in size while defining possible directions for evolutionary change.

## Modulation of morphogen signalling across species

**Figure 2:**
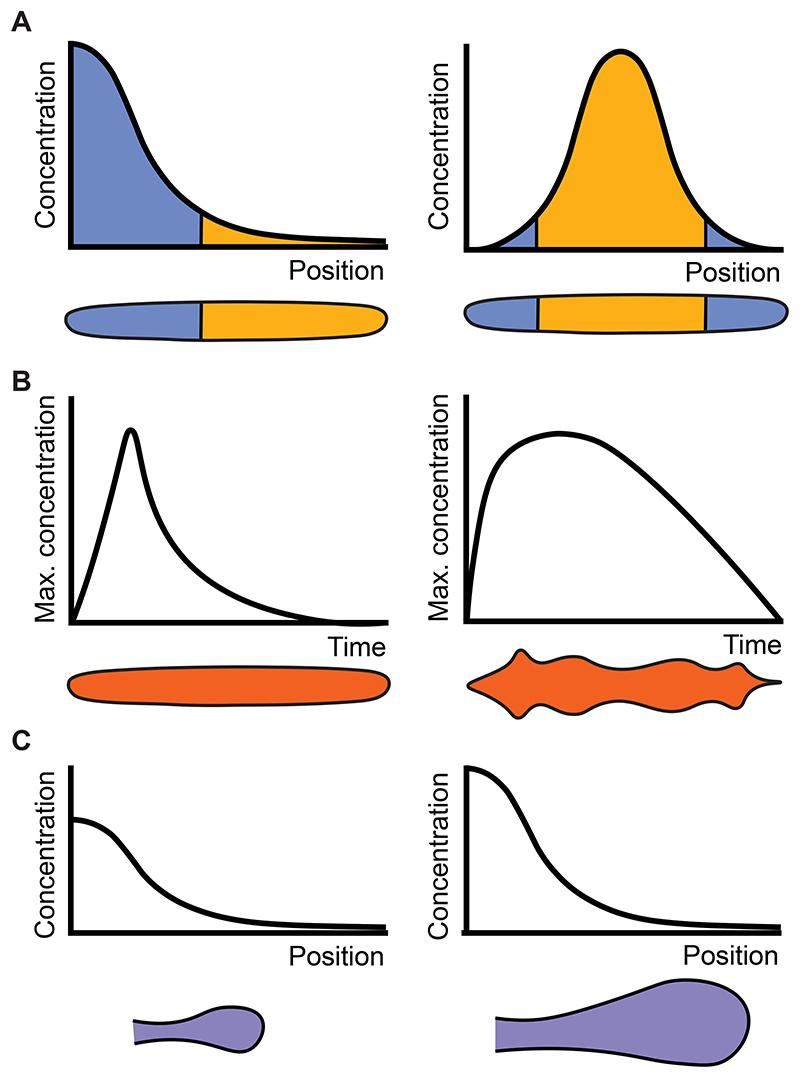
Changes in morphogen expression and dynamics could impact developmental patterning and form. Morphogens prescribe tissue patterning by specifying cell fate boundaries and tissue growth as a function of the spatial or temporal dynamics of morphogen signalling. A: The cartoon illustrates a tissue with a single boundary separating two cell fates (blue and yellow; left) as a response to a specific morphogen gradient. Changing the morphogen spatial expression and corresponding spatial morphogen signal (heterotopy) can lead to changes in the cell fate pattern (right). B: Variation in the temporal dynamics of morphogen signalling (heterochrony) can affect growth and patterning dynamics and the shape and patterning of tissues. The two cartoons indicate putative cases where changes in temporal dynamics in morphogen signalling alter tissue shape. C: Changes in the amplitude of the morphogen profile (heterometry) can influence tissue size, for example when up-regulation of the morphogen concentration leads to increased cell proliferation.

The response of cells to morphogen inputs gives rise to tissue-level patterning and growth, and is both highly varied and context dependent. Examples where cell fate is directly mapped by thresholds in morphogen concentration, and where cells respond to the duration or dynamics of the morphogen input, have all been reported, and the topology of GRNs downstream of morphogens is crucial in defining cellular response (for a review see (Kicheva & Briscoe, 2023)). It follows that, depending on context, variations in the spatial and temporal dynamics of morphogen expression and signalling may affect morphological patterning (Fig. 2). By contrast, the invariance of morphogen-signalling and downstream patterning to noise, changes in size and other perturbations seen in the previous section seems to be at odds with the remarkably diverse forms regulated by the same morphogens across evolution. To illustrate if and how modulation in morphogen signalling may have played a role in the diversification of pattern and form, we summarise examples where variations in morphogen spatial expression pattern and signalling gradient shape (heterotopy, Fig. 2A), temporal dynamics (heterochrony, Fig. 2B) or signalling magnitude (heterometry, Fig. 2C) have contributed to changes in organ size, shape, and pattern throughout evolution. It is now widely accepted that the evolution of morphological diversity is highly dependent on transcriptional regulation that impacts the expression of highly conserved molecular players (Prud’homme et al., 2007). For a review of how GRNs and their molecular underpinnings impact diversification see (Peter & Davidson, 2011). Here, we focus on regulatory changes that are directly associated with the spatiotemporal expression and signalling levels of morphogens.

### Spatial expression of Wnt in the diversification of insect wing pattern

While the familiar *D. melanogaster* has little pigmentation in the wing, the *Drosophila* genus demonstrates wide diversity with a range of black and brown spotted, banded and speckled wing patterns. Comparative analyses between *D. melanogaster* and members of the *D. quinaria* group have elucidated the mechanism of wing pigment evolution and diversification. Wing pigmentation patterns evolved via co-option of the morphogen wingless (Wg), a member of the Wnt family that is crucial for *Drosophila* development (Werner et al., 2010). The gain of an enhancer, *vein spot*, within the cis-regulatory region of *yellow* coupled pigmentation to Wg. Subsequent spatial modulation, or heterotopy, of Wg expression has led to the diversification of wing pigmentation patterns. *Vein spot* is only found in pigmented species, is activated by Wg signalling, and acts to up-regulate production of Yellow, a protein required for melanin production. In pigmented species such as *D. guttifera* and *D. quinaria, wg* spatial expression correlates with areas of pigment and expression of *wg* is sufficient to induce ectopic pigmentation (Werner et al., 2010).

**Figure 3:**
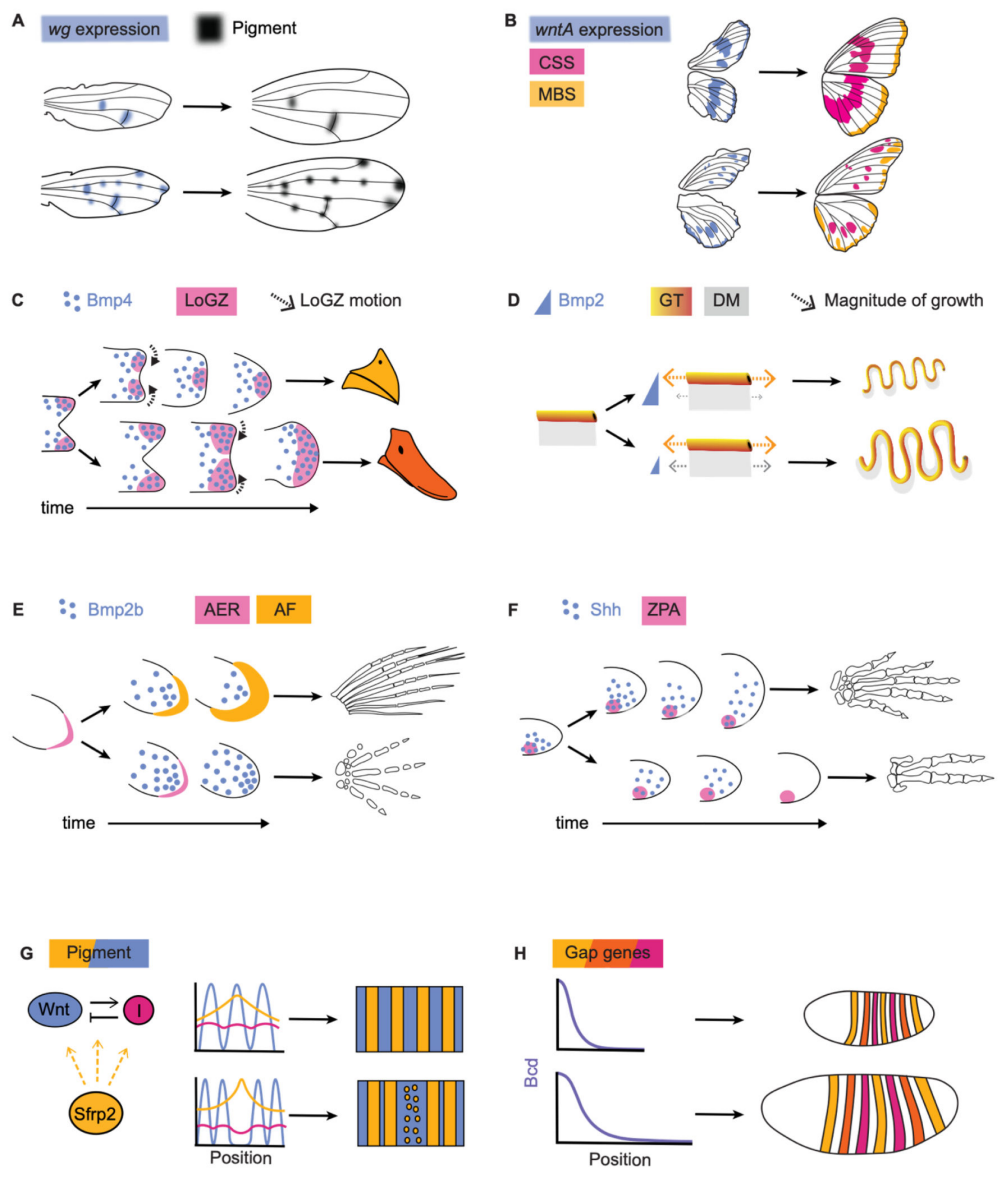
Examples where modulation of morphogen signalling maps to changes in patterning and form between species. A: Spatial modulation of *wg* expression (blue) in developing *Drosophila* wings leads to variation in the spatial distribution of pigment (black), such as between the common ancestor of the *D. quinaria* and *D. virilis* species (upper) and the *D. guttifera* lineage (lower) (Werner et al., 2010). B: In Nymphalidae butterfly wings, WntA delineates the Central (CSS; pink) and Marginal Band Symmetry Systems (MBS; yellow) (Martin & Reed, 2014). Spatial modulation of *wntA* expression (blue) leads to changes in the position and shape of the Symmetry Systems between species. Examples indicative of *Vanessa cardui* (upper) and *Agraulis incarnata* (lower) are shown here (Hanly et al., 2023). C: Bmp4 (blue) specifies the spatiotemporal dynamics of Localised Growth Zones (LoGZ; pink) in the developing beak. Differences in the time of coalescence of LoGZ explain the morphology of the chick’s conical beak (upper) and duck’s broad beak (lower) (Wu et al., 2006). D: In the develop*-ing gut, Bmp2 (blue) expressed in the dorsal mesentery (DM; grey) regulates the growth (arrows) of the DM, thus perturbing the degree of differential growth between the DM and the gut tube (GT; yellow). Changes in Bmp2 expression levels lead to variation in the radius and wavelength of loops, such as between the mouse (upper) and zebra finch (lower) (Nerurkar et al., 2017). E: Increased Bmp2b activity (blue) promotes the persistence of the Apical Ectodermal Ridge (AER; pink) in the developing tetrapod limb (lower), inhibiting its transformation into an Apical Finfold (AF; yellow) and subsequent formation of rays as in the developing fin (upper) (Varga & Varga, 2022). F: Shh (blue) secreted from the Zone of Polarising Activity (ZPA; pink) acts to specify digits in mammals and lizards. Modulation of the spatiotemporal Shh activity affects the number of digits, for example more widespread up-regulation of Shh receptor Ptch1 in the mouse limb (upper) leads to a higher number of digits compared to the bovine limb (lower) (Lopez-Rios et al., 2014). G: Interactions between Wnt (blue) and its inhibitor (I; pink) can drive periodic patterns and underlie coat patterning in rodent species. Modulation of interactions between Wnt and I via the regulator Sfrp2 (yellow) can modulate the length-scale of Wnt expression that in turn impacts coat patterns. This model has been used to recapitulate the morphospace of spots and stripes observed across rodent species (Johnson et al., 2023). H: The decay length of Bcd (blue) scales with embryo size between higher Diptera species, leading to proportional patterning of the gap genes (yellow, orange, pink) between species of different sizes, such as *D. melanogaster* (upper) and *D. busckii* (lower) (Gregor et al., 2005).

Following the co-option of *wg* in colour pigmentation, modulation of the spatial expression of *wg* has contributed to the evolution of different pigmented patterns in *Drosophila* wings (Koshikawa, 2020). For example, the pattern of *D. guttifera* has novel zones of pigmentation in comparison to closely related species such as *D. quinaria*. These zones, found at the campaniform sensilla and longitudinal vein tips, spatially correlate with *wg* expression unique to *D. guttifera* (Koshikawa et al., 2015; Fig. 3A). The novel zones of *wg* expression emerge due to changes in the cis-regulatory sequence of *wg*, thus causing the different spatial pigmentation in the wings of the different species (Karasawa et al., 2023).

Heterotopic expression of *wnts* has also played a role in the diversification of butterfly wing patterning. Colour patterning in the wings of butterflies of the Nymphalidae family has multiple functions such as camouflage, courtship and predator deterrence, and has undergone rapid diversification (Van Belleghem et al., 2021). The butterfly wing pattern consists of multiple independent stripe-like elements, termed symmetry systems (Nijhout, 1994). Between species in the Nymphalidae family, variation in the spatial expression of *wntA* correlates with differences in the shape, size and pigment of three of the four symmetry systems (A. Martin et al., 2012; Fig. 3B). Disruption of WntA signalling leads to a change in the positions of boundaries between black melanated regions and light regions that lack melanin in a dose-dependent manner (Mazo-Vargas et al., 2017). In *Heliconius* butterflies, *wntA* knockouts display a reduction in area of black regions, which are replaced with the red or yellow colour of adjacent regions (Mazo-Vargas et al., 2017). Furthermore, experimental modulation of spatial *wnt* expression reproduces observed natural variation. For example, mosaic *wntA* knock-outs of *H. erato demophoon* result in the loss of black boundaries in areas spatially correlated with the loss of *wntA*; these areas are replaced by expanses of red or yellow, resembling the natural phenotypes of *H. sara* and *H. leucadia* (Mazo-Vargas et al., 2017).

### Spatiotemporal levels of BMP in beak and gut morphogenesis

Avian beak morphology exhibits great diversity in beak size and shape across species (Mosleh et al., 2007). During development, cell proliferation in the developing beak is confined to localised growth zones (LoGZ) within the frontal nasal mass at the tip of the beak (Wu et al., 2006). The number and activity of LoGZ varies between species and is correlated with beak shape. For example, while chickens and ducks both have two lateral LoGZs early in development, the two zones converge at different developmental times (Wu et al., 2004; Fig. 3C). Specification of LoGZs has been linked to Bmp4 levels in the developing beaks of the chick, duck (Wu et al., 2004), cockatiel (Wu et al., 2006) and finch (Abzhanov et al., 2004). Furthermore, spatiotemporal *bmp4* expression correlates with beak width and depth across these species: higher levels of *bmp4* (heterometry) expressed earlier (heterochrony) in development are correlated with deeper, broader beaks (Abzhanov et al., 2004). Injection of Bmp4 throughout the developing chick beak leads to a larger beak, whereas injection of Noggin, a Bmp-antagonist, leads to reduction in beak size (Abzhanov et al., 2004; Wu et al., 2004). In the chick, ablation of the frontal ectodermal zone, a signalling centre that regulates Bmp signalling (Hu & Marcucio, 2009), leads to growth arrest, which can be partially recovered through injection of Bmp4 (Wu et al., 2004). Conversely, injection of Bmp4 into the frontal nasal mass region of the non-ablated beak induces a new growth zone which transforms the chick’s beak to a broad duck-like shape (Wu et al., 2004; Fig. 3C). Furthermore, injection of Bmp4 into chick mesenchyme leads to a broad beak, similar to that of the finch *Geospiza magnirostris*, while injection into the ectoderm produces a sharp beak, similar to that of *Geospiza difficilis* (Abzhanov et al., 2004). These data together suggest that the amplitude, timing and location of Bmp4 signalling impact growth within the developing beak, ultimately influencing beak size and shape.

Theoretical work combined with morphometric quantification across species suggests that feedback between morphogen signalling and tissue geometry in developing beaks controls the dynamics of the LoGZ and induces geometry-driven growth (Al-Mosleh et al., 2021). In this case, higher beak curvature causes cells at the boundary to experience lower morphogen levels, resulting in less cell proliferation. This means that regions of higher curvature are correlated with faster beak depth reduction towards the beak tip, and suggests that feedback between morphogen signalling and tissue architecture impacts growth dynamics and tissue shape during beak development and evolution. Further work is required to establish the spatial profile of cell proliferation, how Bmp4 levels regulate growth in the developing beak and how underlying genetic processes influence *bmp* expression.

Bmp also regulates organ growth and shape during gut morphogenesis (Chevalier, 2022). During development, the straight, tubular midgut undergoes looping which generates a regular, compact structure with high surface area to volume ratio (Chevalier, 2022; Fig. 3D). The gut tube is attached to the abdominal wall via the dorsal mesentery, and differential growth between the gut tube and the dorsal mesentery leads to compressive forces, spontaneous buckling, and the formation of loops (Savin et al., 2011). The morphology of the loops is highly stereotyped within a species, but varies between species (Lavin et al, 2008). These differences in shape are governed by the magnitude of the differential growth between the gut tube and dorsal mesentery, and the geometric and elastic properties of these two tissues. A theoretical model describing this buckling process has accurately predicted the radius of curvature (tightness) and wavelength (size) of the loops given the species-specific growth and mechanical parameters for the mouse, chick, quail and finch (Savin et al., 2011).

Bmp levels regulate both the differential growth between the gut tube and dorsal mesentery and the radial growth of the gut tube (Nerurkar et al., 2017). Increasing Bmp2 activity in chick embryos suppresses the elongation of the dorsal mesentery without affecting the elongation of the gut tube, thus increasing the differential growth between the two tissues. Additionally, increasing Bmp2 activity decreases radial growth of the gut tube. This leads to tighter, smaller loops, that resemble the mouse gut. Vice versa, reduced Bmp2 activity leads to looser, larger loops, closer to the zebra finch gut (Nerurkar et al., 2017). Comparative studies have found that the chick has higher levels of Bmp signalling than the zebra finch; this enhances differential growth between the gut tube and dorsal mesentery and decreases radial growth, thereby explaining the looser, larger loops in the finch (Savin et al., 2011).

Further examples of heterometry and heterotopy in Bmp systems include the evolution of the bat wing from a mouse-like limb. Bats possess dramatically elongated forelimb digits and an interdigital membrane, two anatomical hallmarks of powered flight (Thewissen and Babcock, 1992). During development, bat limbs exhibit higher *bmp2* expression and Bmp signalling in comparison to the mouse, which leads to increased cartilage proliferation and differentiation and results in longer forelimb digits (Sears et al., 2006). In the interdigital space, locally reducing Bmp signalling prevents apoptosis and leads to the maintenance of interdigital webbing (Weatherbee et al., 2006). Furthermore, modulation of Bmp signalling has been linked to changes in cell contractility, proliferation and movement, and may underlie how gastrulation movement have diversified across vertebrates. For example, manipulation of the size and shape of the mesoderm in the chick embryo via Bmp signalling inhibition can produce amphibian-like tissue organisation and flow (Chuai et al., 2023). These studies highlight how morphogen-driven growth and its interaction with tissue mechanics can be modulated in the course of evolution to impact organ size and form.

### Temporal dynamics of morphogens in the fin-to-limb transition and digit evolution

Variation in Bmp signalling may have contributed to the transition from fish fins to tetrapod limbs (Varga & Varga, 2022; Fig. 3E). At the anatomical level, fins and limbs exhibit markedly different bone morphology, but at the genetic level share much of the same developmental machinery (Onimaru et al., 2016). Fin and limb development are both regulated by a distal signalling centre called the apical ectodermal ridge (AER) (Lin & Zhang, 2020). The growth dynamics of the AER differ between fin and limb development: in fins, the AER enlarges and folds into an apical finfold leading to the formation of dermal rays; whereas the AER in the limb does not fold or elongate (Yano & Tamura, 2013). In the absence of this conversion to a finfold, the signalling activity of the AER persists, leading to cell proliferation and eventually digits (Dudley et al., 2002; Fernández Terán & Ros Lasierra, 2008; Fig. 3E). The prevention of finfold formation in zebrafish leads to the downstream expression of genes found in digit formation, which suggests that delay in or absence of AER to finfold conversion and the resulting reduction in the finfold size is a necessary step in the formation of digits (J. Zhang et al., 2010).

Enhancement of Bmp signalling in the fin drives the AER to persist, thus preventing ray formation and enabling the evolutionary transition towards the limb (Cadete et al., 2023; Castro et al., 2021). Increasing *bmp2b* expression via over-expression of *hoxd13a* during zebrafish fin development leads to simultaneous reduction of the finfold and expansion of distal tissue, a phenotype closer to tetrapod digits (Freitas et al., 2012). In zebrafish mutants with expanded finfolds, lower levels of Bmp2b are detected (Castro et al., 2021). Furthermore, finfold length correlates with Bmp levels across zebrafish mutants (Cadete et al., 2023). In the mouse, inhibition of Bmp activity during limb development is associated with the enlargement and persistence of the AER, which subsequently leads to defective digit formation (Cadete et al., 2023). In summary, this evidence establishes modulation of Bmp signalling through upstream factors such as *hox13* as a mechanism to reduce the finfold size, an essential component of the transition from fins to limbs.

Following the evolution of the pentadactylous limb, tetrapod limbs diversified, with multiple instances of digit loss occurring throughout evolution (Saxena et al., 2017). The secreted morphogen Shh has a crucial role in digit number and identity specification and has been implicated in digit evolution (Saxena et al., 2017). In vertebrates, *shh* is expressed in the zone of polarising activity (ZPA) at the posterior margin of the developing limb and forms a gradient along the AP axis (Tickle & Towers, 2017; Fig. 3F). Transplantation of the ZPA to the anterior side of the limb bud leads to a reversal of digit order along the AP axis (Riddle et al., 1993) and digit identity is correlated with the dose of Shh (Yang et al., 1997). Shh also impacts progenitor survival later in development by preventing apoptosis, thus ensuring the presence of sufficient skeletal tissue for digit formation and ultimately impacting digit number (Towers et al., 2008; J. Zhu et al., 2008). Across reptiles and mammals, changes in the spatiotemporal levels of Shh have been linked with variability in digit number and identity (Saxena et al., 2017). Modulation of the Shh receptor Ptch1 has been proposed to underlie the evolution of digit loss in bovines (Lopez-Rios et al., 2014; Saxena et al., 2017). Ptch1 is up-regulated in a smaller region in the bovine limb bud compared to the mouse, which increases the range of Shh and its downstream target Gli1. Inactivation of Ptch1 in the mouse limb bud mesenchyme causes loss of digits similar to the bovine phenotype (Lopez-Rios et al., 2014). This effect is not unique to mammals; inhibition of Shh signal transduction progressively reduces the number of digits in salamanders (Stopper & Wagner, 2007). Furthermore, comparisons between species of *Hemiergis* lizards, with digit numbers ranging from two to five, show that the duration of *shh* expression is shorter in species with fewer digits and corresponds to reduced mesenchymal proliferation and reduction in skeletal elements (Roscito et al., 2015; Shapiro et al., 2003). These results spanning mammals to reptiles suggest that heterochrony and heterometry in Shh signalling duration and magnitude have impacted digit number evolution.

### Morphogen gradient shape in organ patterning and body plan organisation

The periodic colour patterns seen on the skin of many animals have been attributed to morphogens. Rodents, for example, have evolved diverse patterns on their coats, ranging from longitudinal stripes to spots, of varying size, wavelength and colour (Staps et al., 2023). In the striped mouse *Rhabdomys pumilio*, variation in hair length underlies the periodic patterns in coat colour, with shorter hair being darker than longer hair (Johnson et al., 2023). The shorter hairs emerge due to spatially structured delays in hair follicle placode generation during development, which has been attributed to interactions between Wnt and its secreted modulator Sfrp2. The expression of s*frp2* is spatially graded, and abolishing Sfrp2 disrupts patterning. Furthermore, Sfrp2 and Wnt levels are anti-correlated and placodes develop in regions of increased Wnt levels (Johnson et al., 2023). Comparative analysis has shown that striped mice evolved lineage-specific changes in *sfrp2* regulatory elements (Staps et al., 2023). Quantification of the coat patterns across more that 100 species of rodent combined with reaction-diffusion models indicate that modulation of the ranges of activator and inhibitor levels, corresponding to Wnt and Sfrp2, can explain the observed pattern morphospace, including developmental constraints (Staps et al., 2023; Fig. 3G). These studies together provide evidence that modulation of the morphogen range via the regulation of secreted modulators on the cell membrane played a role in the evolution of rodent coat patterning.

In many instances, the same morphogen patterns organs and body-plans that differ markedly in size (Madamanchi et al., 2021). This requires the adaptation of the decay length of the morphogen gradient to remain relevant at the length-scale of different organisms. For example, comparison across *Drosophila* species indicates that the scaling of embryo segments across higher diptera is facilitated by scaling of the Bcd morphogen gradient (Gregor et al., 2005; Fig. 3H). A similar scaling across species was observed during the patterning of the developing neural tube. Comparisons between chick and zebra finch show that scaling of the Shh gradient amplitude, and possibly decay length, together with cell autonomous response to Shh, underlie the scaling of progenitor zones with the size of the neural tube in the two species (Uygur et al., 2016). An open question is whether scaling mechanisms within species discussed in the previous section contribute towards morphogen scaling across species. A potential limitation is the range of sizes such mechanisms can operate over (Ben-Zvi & Barkai, 2010), such that additional mechanisms may be required to explain the cross-species scaling.

### Morphogen-driven diversification: potential and constraints

The examples outlined in this section highlight the capacity of morphogen-mediated patterning and growth for diversification. Earlier, however, we saw that morphogen-mediated patterning and growth is highly robust to changes in size and the expression levels of morphogens and their receptors or other regulators. How can we reconcile these observations? One idea discussed earlier in the article, is that the mechanisms that maintain developmental robustness withstand perturbations up to a given magnitude, but respond discontinuously when pushed beyond a threshold, much like a tipping point (Hallgrímsson et al., 2023). Examples discussed in this review, such as beak and gut development, could be used to directly assess this hypothesis, for example by quantitatively manipulating the levels of over-expression of morphogens and mapping phenotypic response. This offers an avenue to directly and mechanistically assess how specific but highly conserved developmental mechanisms encompass both robustness and evolvability.

We have focused on instances where morphogens act primarily as growth or patterning factors, but morphogens often hold a role in both patterning and growth control in the same developmental context. In principle, when morphogens guide both patterning and growth mutations that affect their spatiotemporal dynamics can simultaneously impact both size and morphological patterning. While this could imply developmental constraints that limit evolution, it may also lead to diversification, for example when selection for a variation in size leads to variation in spatial patterns, introducing new morphological designs. Comparative studies can be employed to assess this question by exploring the degree to which organ size and pattern remain correlated across species and conditions under which such correlations break down.

Overall, we understand little about how specific morphogen mechanisms constrain or facilitate evolutionary change. Although the comparative studies summarised here imply that morphogen-driven patterning can underpin evolutionary change, the types of mutations driving these changes, the ways in which standing variation is generated and exploited in populations and the potential evolutionary dynamics for these evolved features are not known. Exploring the capacity of morphogen-driven patterning for diversification in synthetic and experimental evolution settings can shed light on these questions (P. Li et al., 2018; Santos-Moreno et al., 2023; Stapornwongkul et al., 2020). In addition, theoretical frameworks can be used to interrogate the genotype-to-phenotype map in the context of specific patterning mechanisms (Al-Mosleh et al., 2021; Staps et al., 2023). An important consideration here is the feedback mechanisms that allow patterning and growth to remain resilient to perturbations, as well as the architecture of the regulatory networks that interpret morphogen inputs.

## Theoretical tools for understanding the evolution of development

Directly investigating the evolution of development in multicellular organisms is challenging, both due to the complexity of developmental processes and the long timescales required to observe morphological evolution. Advancements in experimental evolution and synthetic biology are generating new insights into this field. For example, the evolution of morphogen-driven patterning has recently been explored using synthetic GRNs downstream of a morphogen (Santos-Moreno et al., 2023). This framework has been used to study pattern robustness and evolvability, and properties of the genotype-to-phenotype map. Furthermore, recently developed tools utilising synthetic morphogens *in vitro* (P. Li et al., 2018) and *in vivo* (Stapornwongkul et al., 2020; Toda et al., 2020) offer a powerful framework to investigate design principles of morphogen gradient formation, as well as pattern and size regulation. Alternatively, laboratory evolution experiments leveraging organisms with shorter lifespans can directly interrogate genetic and phenotypic changes over generations. For instance, in recent work, parallel evolution experiments on fly embryos were performed to investigate the adaptive response to changes in *bcd* dose (X. C. Li et al., 2022). The increasingly high-throughput capabilities of these types of experimental techniques, such as automated imaging and whole-genome sequencing for parallel evolution experiments, offer a promising avenue towards distilling the principles and constraints that underlie developmental patterning and its evolution. Coupling theoretical frameworks, such as biophysical models for morphogen and GRN dynamics, to these approaches could prove invaluable for mechanistic interpretations of experimental observations, to explore the evolutionary implications of their results, and to form new hypotheses and the means to test them. Phase space analysis, mutational studies and evolution algorithms have all been used to assess how specific developmental mechanisms may have evolved and diversified. Here, we give a brief overview of these methods.

**Figure 4:**
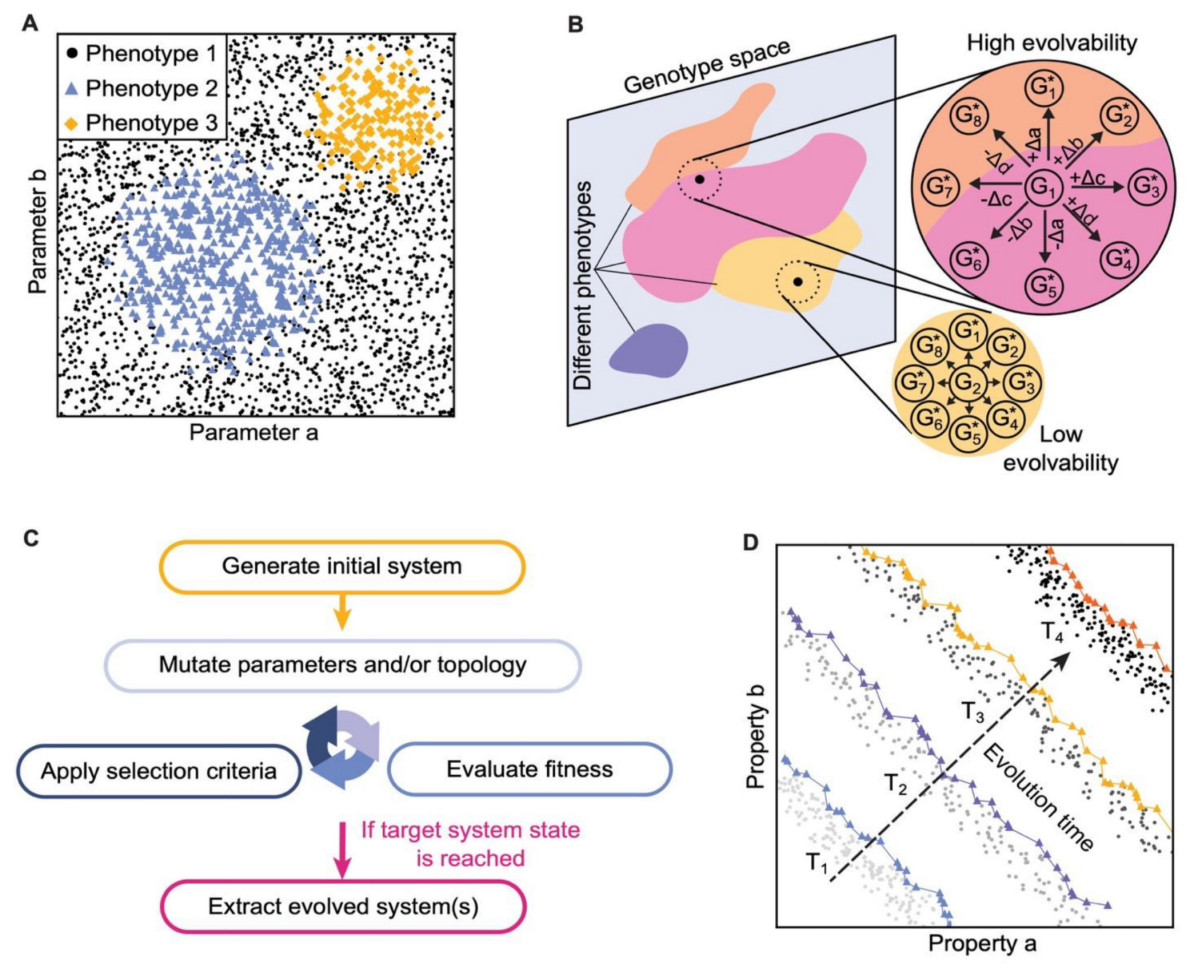
Theoretical methods for studying the evolution of development. A: Schematic example of a phase space analysis where two system parameters (a and b) are varied over significant ranges, and the areas where specific criteria or phenotypes are met are identified (blue, yellow and black). The parameters varied represent the ‘genotypes’, and can in theory be sampled from a high-dimensional phase space. B: Mutational studies explore how specific networks or mechanisms respond to mutation. Different colours represent different phenotypes accessible by varying the genotype. Genotypes that can access multiple phenotypes following a single mutation in any system parameter are highly evolvable (top right). Here, varying hypothetical system parameters a, b, c, d by small amounts Δa, Δb, Δc, Δd respectively lead to new genotypes G* that correspond to different phenotypes (pink, orange). These systems have reduced mutational robustness. Genotypes that cannot access multiple phenotypes (bottom right) are less evolvable as multiple mutations are required to change their phenotype. C: The general structure of an evolution algorithm. Following the generation of an initial system and its associated parameters, parameters are mutated and the system fitness is evaluated. Evolution is complete when the system reaches a pre-defined fitness threshold or when a mutation-selection balanced is reached so that no further improvement in fitness can be achieved. D: During Pareto evolution, the Pareto front (coloured triangles) represents the systems for which no property of interest (for example the hypothetical properties a and b) can be improved without worsening another. Pareto fronts are labelled for four different times during evolution (T_1,2,3,4_), with the arrow indicating the direction of system evolution perpendicular to the Pareto front. The values of properties are improved throughout evolution, and the final Pareto front (orange) defines the final co-optimised value of each system property at the end of evolution.

Phase space analysis involves sweeping through a large number of parameter values or gene network topologies and measuring how these influence system function (Adler et al., 2017; Cotterell & Sharpe, 2010; Fig. 4A). For example, this method has been used to identify network motifs that can generate ‘stripes’ of gene expression in morphogen patterning systems and has obtained the motifs observed experimentally during both gap gene expression in *Drosophila* and mesoderm induction in *Xenopus* embryos (Cotterell & Sharpe, 2010). Mutational studies take this one step further by exploring what is mechanistically and phenotypically attainable through mutation (Jiménez et al., 2015; O. C. Martin & Wagner, 2008; Santos-Moreno et al., 2023; Fig. 4B). This method is distinct from phase space analysis in that it explores the local neighbourhood of given parameters and network topologies for a system, which is useful for investigating the evolvability of specific mechanisms and for quantifying mutational robustness.

Instead of randomly sampling the entire parameter space for possibly rare, high-fitness network designs, evolution algorithms directly incorporate the processes of mutation and selection to study how new phenotypes or functionalities emerge (François, 2014). Evolution algorithms have been compared to ‘forwards genetic screens’, since they can generate the mechanisms responsible for different phenotypic traits (Warmflash et al., 2012). One approach is to explore the ‘genotype’ space by using Markov Chain Monte Carlo (MCMC) methods to generate a biassed random walk according to a target distribution for a specific feature or phenotype (Burda et al., 2011; Hernández et al., 2022; Fig. 4C). Other evolution algorithms instead follow the evolution of a large ‘population’ of genotypes, where selection and duplication steps incorporate reproduction and inheritance (for reviews see (Deb, 2011; François, 2014)). Evolution algorithms generally out-perform phase space analysis in locating rare, high-fitness network designs due to the large number of relevant parameters and the clustering of viable networks in parameter-space (Ciliberti et al., 2007; O. C. Martin & Wagner, 2008).

To what extent these evolution algorithms recapitulate mutation and selection in real populations is not clear. One challenge is the definition of selection and fitness functions and the way these recapitulate fitness in real populations (François & Siggia, 2008). Nonetheless, evolution algorithms are useful theoretical frameworks for exploring how a phenotype space can be navigated given the specific set of rules or mechanisms that define it. For example, evolution algorithms have been used to probe the *de novo* evolution of morphogen-mediated patterning (François, 2014). At the single cell-level, evolution algorithms have been used to derive networks that can act as bistable switches, oscillators, perfect adaptors or adaptive sorters; these networks are comparable to those predicted to control developmental processes such as *Xenopus* oocyte maturation and circadian networks in *Drosophila* (François & Hakim, 2004; François & Siggia, 2008; Lalanne & François, 2013). At the tissue-level, evolution algorithms have also been used to obtain ‘optimal’ regulatory networks that qualitatively reproduce the gap gene expression profiles observed in *Drosophila* (Sokolowski et al., 2023), and to compare network designs that could drive segmentation in short versus long germ-band insects (François & Siggia, 2010; François et al., 2007). Future efforts to allow direct comparison between laboratory evolution and evolution algorithms could help explain how specific but highly conserved mechanisms, such as morphogen- and GRN-driven patterning, impact evolutionary dynamics and outcomes.

Finally, when multiple desirable features entail trade-offs, evolution algorithms can implement the principle of Pareto evolution to identify co-optimised parameter values and network topologies (Deb, 2011; Starr, 2011). In this case, mutations are accepted if they improve at least one target property without worsening any other, defining a Pareto front (Adler et al., 2017; Szekely et al., 2015; Warmflash et al., 2012; Fig. 4D). Pareto evolution has been used to investigate why specific network motifs are evolutionarily favoured (Adler et al., 2017; Burda et al., 2011), as well as to probe how effective fitness functions can be used to capture the simultaneous optimisation of multiple features (Henry et al., 2018).

## Conclusion

A key challenge in the study of the evolution of development is to reconcile the highly non-linear, multiscale mechanisms that control developmental patterning and growth with the processes of mutation and natural selection. This missing link is fundamental for understanding the forces and mechanistic constraints that guide evolution. For example, the stochastic nature and temporal progression of mutation and ecological changes that lead to fitness shifts can contribute towards the trajectory of evolution by restricting the space of locally accessible genotypes and therefore morphologies. In addition, the architecture of developmental programs and physical constraints have long been recognized as setting limits on the phenotypes that are biologically feasible. The significance of these factors is extremely challenging to disentangle with experiments alone. This is in part due to the complexity that underlies the process of development, but also the timescales required to observe morphological evolution in complex organisms.

One way forward is to focus on specific, mechanistic and evolutionarily-conserved mechanisms for patterning and growth, and to use a combination of theoretical and experimental approaches to investigate how change may occur during evolution. Morphogen-mediated patterning and growth are hallmarks of development, are highly conserved across species, and offer a malleable system that can be investigated *in vivo*, in synthetic contexts, and using theoretical models. In this Review, we have summarised examples of comparative studies that show how changes in morphogen-mediated patterning and growth have played a role in the diversification of patterning and form. We argue that developing appropriate theoretical frameworks to be used alongside comparative studies, laboratory evolution experiments and synthetic patterning methods could help elucidate the potential of these conserved mechanisms for pattern diversification, as well as what constraints they entail. Such frameworks can be expanded to investigate more general hypotheses about the evolution of development and can be applied in other contexts such as to the process of morphogenesis and its associated potential mechanical constraints. In conclusion, the field has reached an exciting point, with rapid computational advancements and new experimental tools providing a fertile ground for studies that can deepen our understanding of how development evolves.

## Summary statement

We review how morphogen-mediated patterning and growth evolve, the mechanistic constraints that underlie this diversification, and the ways in which theory and experiment can be interwoven to bring new perspectives to the field.

## Glossary

### Abbreviations

Co-option: the mechanism whereby a gene or trait involved in development gains an additional function and is redeployed in a new developmental context
Evolvability: the capacity of a given system to generate adaptive change
Gene regulatory network (GRN): A set of genes or transcription factors that interact with each other to regulate protein expression levels that ultimately determine cell fate
Heterochrony: a change in the timing, rate or duration of developmental events
Heterometry: a change in the magnitude or amount of gene expression during development
Heterotopy: a change in the spatial arrangement or layout of development
Morphogen precision: The ability of a morphogen gradient to generate sharp gene expression boundaries, particularly in noisy environments
Morphogen robustness: The invariance of a morphogen gradient to perturbations in system parameters such as the production rate of the morphogen or its receptors
Morphogen scaling: The adjustment of a morphogen gradient to maintain its spatial proportions when tissue length changes, either during growth, between individuals of a species, or between different species
Neutral mutations: Changes in the genotype that do not impact phenotype or fitness, and thus do not affect selection
Plasticity: the capacity of a given genotype to produce multiple phenotypes in response to variation in the environment
Pareto evolution: An evolution algorithm that accepts mutations only when they cause a number of pre-determined system properties to all either improve or stay at the same level they were before the mutation
